# Factors affecting medical students’ intention to use Rain Classroom: a cross-sectional survey

**DOI:** 10.1186/s12909-024-05037-6

**Published:** 2024-01-24

**Authors:** Hui Lv, Jinghong Low, Siow-kian Tan, Lingjiao Tang, Xuebin Li

**Affiliations:** 1 Faculty of Management, Multimedia University, Cyberjaya, Malaysia; 2 The Affiliated Hospital of Youjiang Medical Universily for Nationalities, Baise, China; 3 College of Nursing, Youjiang Medical University for Nationalities, Baise, China; 4 Modern Industrial College of Biomedicine and Great Health, Youjiang Medical University for Nationalities, Baise, China; 5 School of Economics and Management, Xiamen University Malaysia, Sepang, Malaysia

**Keywords:** UTAUT, Rain classroom, Behavior intention, Medical students

## Abstract

**Background:**

Rain Classroom was one of the most popular online learning platforms in Chinese higher education during the pandemic. However, there is little research on user intention under the guidance of technology acceptance and unified theory (UTAUT).

**Objective:**

This research aims to determine factors influencing students' behavioural intention to use Rain Classroom.

**Methods:**

In this cross-sectional and correlational investigation, 1138 medical students from five medical universities in Guangxi Province, China, made up the sample. This study added self-efficacy (SE), motivation (MO), stress (ST), and anxiety (AN) to the UTAUT framework. This study modified the framework by excluding actual usage variables and focusing only on intention determinants. SPSS-26 and AMOS-26 were used to analyze the data. The structural equation modelling technique was chosen to confirm the hypotheses.

**Results:**

Except for facilitating conditions (FC), all proposed factors, including performance expectancy (PE), effort expectancy (EE), social influence (SI), self-efficacy (SE), motivation (MO), anxiety (AN), and stress (ST), had a significant effect on students' behavioural intentions to use Rain Classroom.

**Conclusions:**

The research revealed that the proposed model, which was based on the UTAUT, is excellent at identifying the variables that influence students' behavioural intentions in the Rain Classroom. Higher education institutions can plan and implement productive classrooms.

**Supplementary Information:**

The online version contains supplementary material available at 10.1186/s12909-024-05037-6.

## Introduction

Countries have abandoned traditional education to combat the spread of the new coronavirus disease (COVID-19) [1]. Millions of students and educators worldwide have been affected, and various e-learning techniques have been deployed to promote learning in the epidemic age [2, 3]. The education system worldwide has begun looking for a new method of education. This has increased the use of e-learning, including MOOCs, Google Meet, Microsoft Teams, Zoom, GoToMeeting, and WebEx worldwide [4]. Higher academic institutions actively respond to the call of China's Ministry of Education (MoE) by suspending physical lessons and launching virtual classes such as MOOC and Rain Classroom. During the ongoing "lockdown" of the pandemic, these digital learning tools were crucial for carrying out education [5].

The Rain Classroom mobile app, created in 2016 in collaboration with the Online Education Office of Tsinghua University, is one of the most widely used electronic learning tools. Its purpose is to integrate teachers and students through smart terminals, give students new experiences before, during, and after class, improve teaching and learning, and push for education reform. [6]. Students can quickly enter the Rain Classroom by entering the lesson code on the projection screen or scanning the QR code displayed there using WeChat, China's most popular social media app [4].

Educators can share MOOC videos, coursework, and exercises with students via Rain Classroom before class, allowing communication and student feedback. Teachers can review lessons at any time, including preview time, duration, response rate, and preview exercise accuracy. The course facilitates teacher-student interactions, including sign-in, synchronized educational courseware, classroom testing, bullet screen interaction, red envelopes, multiscreen interaction, real-time instructional feedback, and classroom submissions. Students receive reports and general PowerPoint slides on their phones after class. In addition, teachers have access to teaching resources, student attendance, student performance, student participation in learning activities, answer analysis, answer details, class statistics, and data regarding the learning process. These resources can be utilized for educational improvement and reflection.

Although domestic researchers and teachers favour Rain Classroom, it is not as well known globally as Google Meet or Microsoft Teams are for similar online learning platforms [4].To successfully implement Rain Classroom in higher education, it is imperative that it is accepted. As a result, it is critical to comprehend and pinpoint the crucial elements that influence Rain Classroom acceptance. However, little research has been done on adopting and receiving Rain Classroom [4, 7].

In previous studies, researchers have used various technology acceptance models, particularly the UTAUT2 and the Technology Acceptance Mode, to identify the elements that affect users' adoption of specific information systems [8–10]. The UTAUT, which is typically used in fields such as e-learning systems [11], MOOCs [12], and health information systems [13], has recently been applied in the education sector to identify factors that affect user-specific acceptance of educational technologies [14].

Having to understand user acceptance, few researchers have attempted to apply the UTAUT paradigm to Rain Classroom (Yang & Yu, 2022) [22]. These studies have yet to uncover all the fundamental components that might have a significant impact on the behavioural intentions of students using Rain Classroom. Having to identify the variables that influence students' behavioural intentions toward Rain Classroom, this study combines the UTAUT with four new external constructs: self-efficacy (SE), motivation(MO), stress(ST), and anxiety(AN).

### Review of the literature

**Fig. 1:**
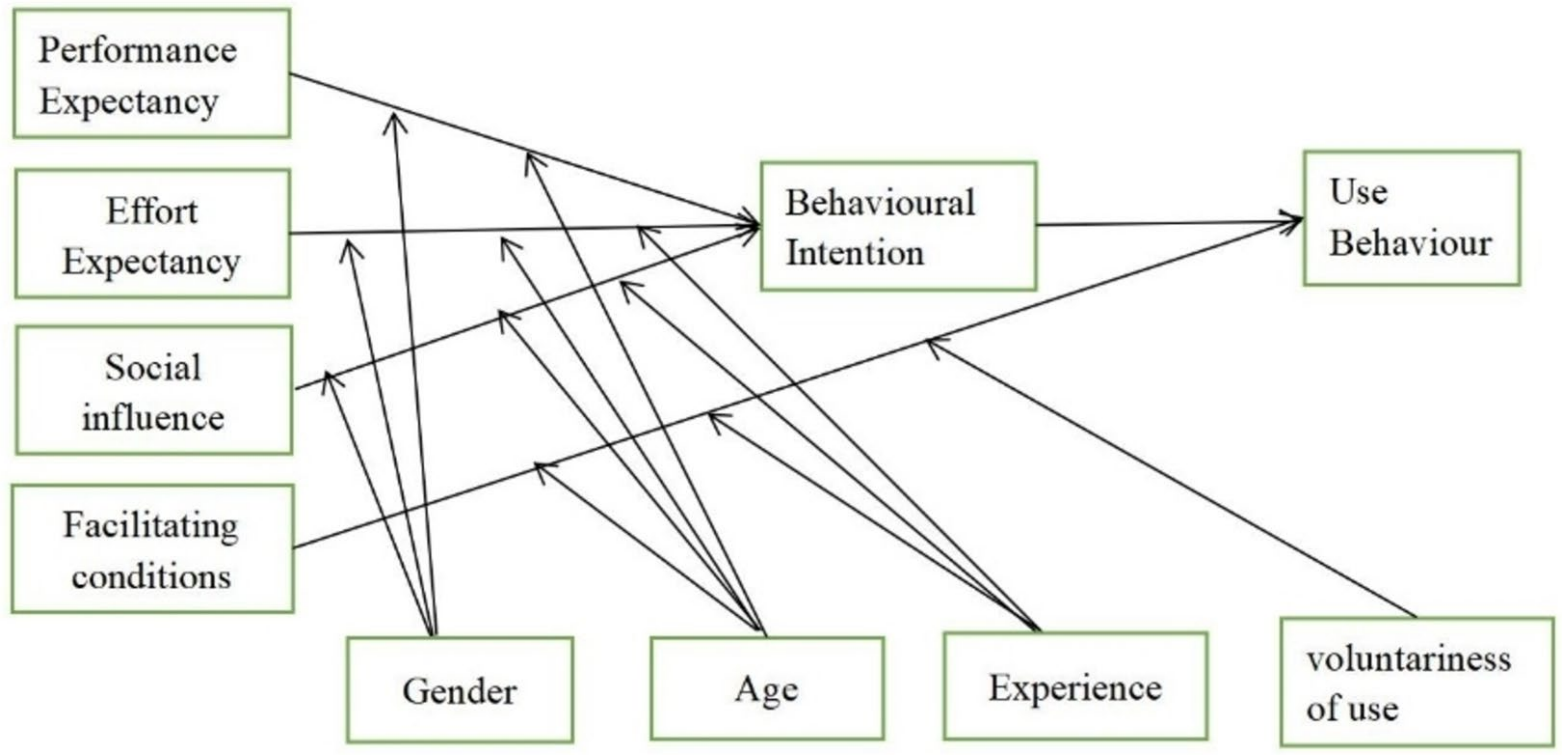
UTAUT model

Researchers have used human behaviour theory for many years to examine the intention of acceptance and use [15]. Various theories have been proposed to explain technology acceptance, including Theory of Reasoned Action (TRA), Theory of Planned Behavior (TPB), Social Cognitive Theory (SCT), Technology Acceptance Model (TAM), Extended Technology Acceptance Model (TAM2), Motivational Model (MM), Model of PC Utilization (MPCU), Innovation Diffusion Theory (IDT), and Unified Theory of Acceptance and Use of Technology (UTAUT)) [15–17]. The UTAUT, which combines eight of these theories, has been used to validate learners' behavioural intentions(Venkatesh et al., 2003). This model consists of four primary constructs: performance expectancy, effort expectancy, social influence, and facilitating conditions (Fig. 1) [18]. The UTAUT is among the most popular theoretical models for predicting technological acceptance and adoption. [17, 19]. The UTAUT model, which had an adjusted R2 of 69% and was superior to the eight individual models, could account for up to 70% of the variance in usage behaviour variance [18]. Research reveals that the UTAUT is a dependable model for understanding global technology adoption factors (Arifet al., 2018). As a result, the UTAUT serves as the theoretical foundation for our investigation. After reviewing the literature, the following relationships were proposed as hypotheses.

### Performance expectancy

PE can be described as a student's level of optimism about using Rain Classroom to enhance academic achievement [18]. Some research has shown the positive impact of PE on behavioural intention [4, 20–22]. A pupil will have a favourable attitude in regard to the system that is being discussed and be more likely to use Rain Classroom if they anticipate improving their academic performance. Thus, the following is a statement of the hypothesis.

- H1: Performance expectancy has a positive impact on medical students' behavioural intention to use Rain Classroom.

### Effort expectancy

EE is the degree to which an individual perceives using a particular technology as easy or difficult [18]. This construct is analogous to the "ease-of-use" component of the Technology Acceptance Model [10]. Research has shown that EE can be a significant predictor of an individual's intention to use an online educational system [4, 21–24]. A meta-analysis of 52 studies found that ease of use had the strongest relationship with users' intentions to adopt new technologies [25]. This data allows us to provide a working hypothesis.

- H2: Effort expectancy has a positive impact on medical students' behavioural intention to use Rain Classroom.

### Social influence

SI refers to a student's perception of others' beliefs about their use of Rain Classroom (Venkatesh et al., 2003), which has been found to positively impact their behavioural intentions toward technology adoption in the workplace. Numerous studies have shown that SI significantly influences the behavioural intention to adopt online learning systems [24, 26–28]. C-M Chiu and ET Wang [29] suggested that a more favourable social influence on a behaviour, such as using an online course system, increases the likelihood of students carrying it out [30]. Hence, we postulate the hypothesis:

- H3: Social influence has a positive impact on the behavioural intention to use Rain Classroom.

### Facilitating conditions

The level of FC indicates a student's belief in the existence of an administrative and technological framework to enable the usage of an online course management system (Venkatesh et al., 2003). According to Venkatesh et al. (2003), FC has a favourable impact on how often people use technology at work. In this regard, many studies have indicated that FC has a considerable influence on users' intentions to use digital information systems [4, 15, 27, 28]. However, some research reports have indicated that FC has no significant impact on their intentions to use e-learning [24, 26, 31]. Based on what we already know, we have come up with the research hypothesis.

- H4: Facilitating conditions have a positive impact on the behavioural intention of medical students to use Rain Classroom.

### Self-efficacy

SE is the degree to which one believes in their ability to complete a task [32]. According to several studies, self-efficacy is one of the important factors determining the acceptance of an educational system [33–35]. Several studies have endorsed the SE variable as an integral part of promoting the adoption of MOOCs by students [16, 21, 36, 37]. These findings contribute to the extension of the UTAUT model related to the construct of SE on computers. Based on this, the study posits the following.

- H5: Self-efficacy has a favorable impact on medical students' behavioral intention to use Rain Classroom.

### Motivation

Self-determination theory (SDT) is a theory that explains human behaviour and motivation [38]. It focuses on fulfilling basic psychological needs and improving intrinsic motivation for learning [39]. The theory categorizes motivation into autonomous and controlled based on the level of autonomy or control [38]. Therefore, by integrating SDT and UTAUT, this study can address the limitations and improve the accuracy and comprehensiveness of its findings. Autonomous motivation, which meets individual autonomy needs, is a critical driver of behaviour [31]. Therefore, we assume the following:

- H6: Motivation has a positive effect on medical students' behavioural intention to use Rain Classroom.

### Stress

According to definitions, ST refers to a specific interaction between an individual and their surroundings in which the individual is draining or exceeding the environment's resources and endangering their wellbeing. [40]. Online learning has been used to assess the daily course and academic performance of students, and in some ways, students felt stressed [41]. MA Islam, SD Barna, H Raihan, MNA Khan and MT Hossain [42]noted that the pressure of online lecture tasks is a contributing factor to students' ST levels. These tasks require students to use online media that they may not be familiar with, leading to the need for immediate comprehension. As a result, the study proposes the following.

- H7: Stress has a negative impact on medical students' behavioural intentions to use Rain Classroom.

### Anxiety

AN is a negative emotional reaction that adversely affects a person's intention to perform a particular task [43]. In this study, task anxiety is defined as the fear of being unable to accomplish tasks [44]. The term "technology AN" refers to people's anxiety or unease when using computers or other forms of technology. [45]. Empirical research has indicated that anxiety directly affects behavioural intention negatively [46–48]. Some research has demonstrated the importance of predicting use behaviour [49]. Therefore, we propose the following hypothesis:

- H8: Anxiety has a negative impact on the behavioural intention of medical students to use Rain Classroom.

## Methods

### Study design

This study utilized purposive sampling because defining the Rain Classroom user population is challenging. To the researcher's knowledge, no comprehensive census or complete list of all Rain Classroom exists in Guangxi, China. We developed a fitting model using a causal research design and verified the hypothesis proposed to understand students' behavioural intention to use Rain Classroom. An online survey platform was used to create and distribute a self-administered questionnaire. Confirmatory factor analysis and structural equation modelling were used to perform additional data analysis. The next section of the methodology is for participants, research instruments, and research procedures. To forecast how medical students will use Rain Classroom, a hypothetical model that uses the UTAUT was created, and its fit and validity were evaluated.

### Participants

The selection of participants was based on four Inclusion criteria and five exclusion criteria to ensure they fulfilled the study's requirements. Inclusion criteria: current enrollment in medical university, smartphone users aged 18 years or above, and willingness to participate.Exclusion criteria: Students not enrolled in a medical course or program at a medical university in Guangxi Province, lack smartphone access, have no prior experience with Rain Classroom, and are underage and non-consenting individuals. The target participants were junior college, undergraduate and postgraduate students from five public medical universities of Guangxi in China, namely, Youjiang Medical University for Nationalities (YMUN), Guangxi Medical University(GXMU), Guangxi University of Chinese Medicine(GXUCM), Guangxi University of Science and Technology (GXUST), and Guilin Medical University(GLMU). These students were studying various medical disciplines, such as clinical medicine, nursing, and foundation medicine, and the students were currently using Rain Classroom.

### Measurement

**Fig. 2:**
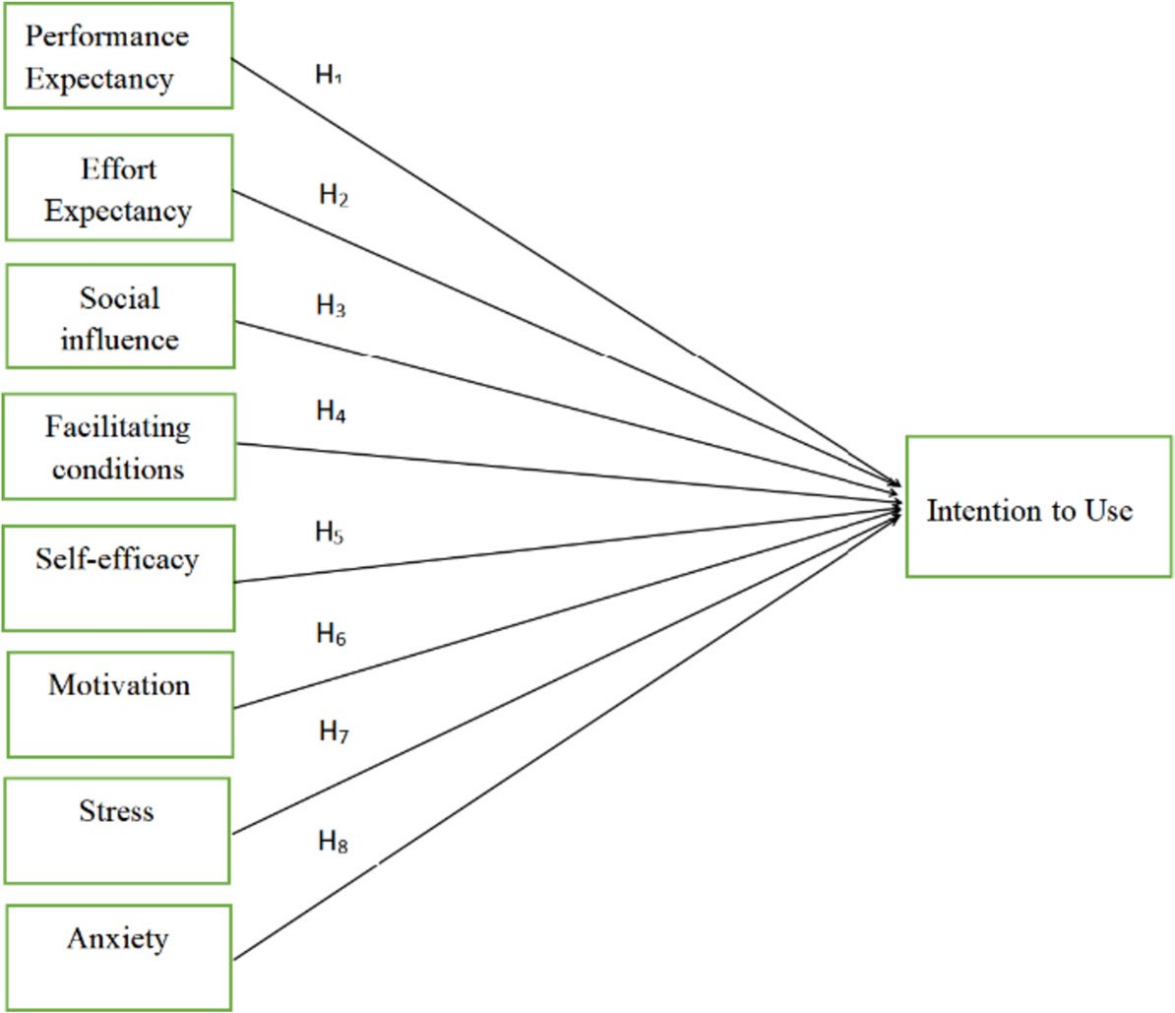
Research model

Utilizing Sojump, an online questionnaire was distributed to students at five medical universities with the objective of data collection. The survey was bifurcated into two sections. The initial section gathered demographic details, while the latter was designed to accumulate data on the research model, encompassing nine constructs with a total of 31 elements (Fig. 2 and Table 2). The constructs included PE, EE, SI, FC, SE, MO, ST, AN, and BI. To bolster the content validity of the questionnaire, all items were adapted from [18, 32, 41, 43]and were subsequently rephrased to align with the context of the Rain Classroom. These included PE (3 elements), EE (3 elements), SI (3 elements), FC (4 elements), SE (3 elements), MO (4 elements), ST (3 elements), AN (5 elements), and BI (3 elements). Participants were instructed to appraise each item employing a five-point Likert scale ranging from 1 (representing strong disagreement) to 5 (representing strong agreement). This measurement technique facilitated participants in expressing their degree of concurrence or discordance with each item on a graded scale, affording a more intricate comprehension of their attitudes and opinions. The reliability of the scales utilized in the study was evaluated using Cronbach's alpha coefficients, exceeding the acceptable threshold of 0.70 [50], thereby indicating the robust reliability of the measures.

### Data collection

The research used a questionnaire survey to implement the quantitative method. The data were collected from December 6, 2022, to February 7, 2023, by distributing self-administered online questionnaires in Sojump. In total, 1,074 questionnaires were distributed to students. For missing values, 35 questionnaires were discarded. Therefore, 1138 questionnaires were incorporated into the preliminary analysis, and the response rate was 97.02%. According to Hair et al. (2010), the minimum sample size for quantitative research is 354. Therefore, the sample size of this study (*N* = 1138) is sufficient. The data of the participants are shown in Table 1.

**Table 1:** Demographics of the participants (*n* = 1138)

Measure	Items	Frequency (%)or Mean ± SD
Sex	Male	280(24.6)
	Female	858(75.4)
Age		20.09 ± 1.527
University	YMUN	553(48.6)
	GXUCM	253(22.2)
	GLMU	159(14)
	GXMU	87(7.6)
	GXUST	86(7.5)
Educational level	Postgraduate	88(7.7)
	undergraduate	772(67.8)
	Junior college	278(24.4)
Daily nonacademic internet use		3.85 ± 1.281
Daily time using Rain Classroom		1.76 ± 984
Purpose of internet use (*n* = 5499)	Communication	1102(20.00)
	Recreation	1033(18.80)
	Learn knowledge	1010(18.40)
	Shopping	969(17.60)
	Obtain information	950(17.30)
	Other	435(7.90)

^a^Multiple responses

### Data analysis

The accumulated data underwent meticulous analysis using the SPSS 21.0 and AMOS 26.0 software suites. The descriptive statistics methodology was leveraged to investigate the intrinsic characteristics of the data set in conjunction with the application of suitable tests designed to evaluate the reliability and validity of the deployed research instrument. A path analysis was conducted to ascertain the compatibility of theoretical models with the behavioural inclinations of medical students towards Rain Classroom usage. The effectiveness and path coefficient estimates of the model were subsequently calculated. An array of indices, including X^2^/df, RMSEA, GFI, NFI, IFI, CFI, PNFI, PCFI, and PGFI, were utilized to assess the model's fit [39]. The structural equation modelling technique was applied to evaluate the stipulated hypothesis. The p-value, used to denote significance, was ascertained to be *P* < 0.05.

## Result

### Features of the population

Of the 1138 participants involved in the study, 858 were women (75.4%), and 280 were men (24.6%) (Table 1). The mean age was 20.07 years. The participants came from five universities, including YMUN(*n* = 553,48.6%), GXUCM(*n* = 253,22.2%), GLMU(*n* = 159,14%), GXMU(*n* = 87,7.6%), GXUST(*n* = 86,7.6%). The majority of the students were undergraduate students (*n* = 772, 67.8%), followed by postgraduate students (*n* = 88, 7.7%) and junior students (*n* = 278, 24.4%). The average time spent on the internet for nonacademic purposes was 3.85 h, for using Rain Classroom was 1.76 h, and the use of the internet was mainly for communication, followed by Recreation, Learning knowledge, Shopping, Obtaining information, and Others.

### Measurement model

This section scrutinizes the evaluation of measurement scales, focusing specifically on item reliability, internal consistency reliability, convergent validity, and discriminant validity [51]. In order to evaluate item reliability, the loadings of each item on its corresponding latent variable are examined, with an emphasis on standardized loadings of at least 0.50, preferably higher than 0.70, and statistical significance for all items [52]. Composite reliability (CR) is a common method to assess internal consistency, referencing a value of 0.70 or higher for each latent variable. Convergent validity is measured through average variance extracted (AVE) testing, requiring a value of 0.50 or higher for each latent variable. Sufficient reliability and convergent validity are reflected in measures that exhibit standardized factor loadings exceeding 0.70 (*p* < 0.001), AVE surpassing 0.50 (*p* < 0.001), and CR greater than 0.70 (*p* < 0.001) (Table 2). Discriminant validity, representing the significant difference between two factors [53], is assessed by scrutinizing the AVE of the UTAUT scale using the method proposed by C Fornell and DF Larcker [54]. The bold diagonal values in Table 3 indicate that the square root of AVE for each variable substantially exceeds the correlation with other variables, signifying the discriminant validity of the constructs and the superior performance of the questionnaire.

**Table 2:** Analyse of reliability and convergence validity

Construct	Code	Factor Loading	Cronbach'sα	CR	AVE
PE	PE1	0.781		0.827	0.614
	PE2	0.778	0.825		
	PE3	0.792			
EE	EE1	0.758		0.806	0.580
	EE2	0.737	0.806		
	EE3	0.789			
SI	SI1	0.795		0.833	0.625
	SI2	0.817	0.833		
	SI3	0.758			
FC	FC1	0.811	0.839	0.842	0.571
	FC2	0.747			
	FC3	0.738			
	FC4	0.723			
SE	SE1	0.792	0.817	0.817	0.599
	SE2	0.772			
	SE3	0.757			
MO	MO1	0.753	0.845	0.845	0.578
	MO2	0.767			
	MO3	0.728			
	MO4	0.791			
ST	ST1	0.833	0.865	0.866	0.683
	ST2	0.799			
	ST3	0.847			
AN	AN1	0.766	0.871	0.872	0.577
	AN2	0.748			
	AN3	0.725			
	AN4	0.723			
	AN5	0.831			
BI	BI1	0.743	0.814	0.813	0.592
	BI2	0.790			
	BI3	0.775

**Table 3:** Discriminant validity results

	**PE**	**EE**	**SI**	**FC**	**SE**	**MO**	**ST**	**AN**	**BI**
PE	**0.784**								
EE	0.385	**0.762**							
SI	0.381	0.495	**0.790**						
FC	0.424	0.356	0.322	**0.756**					
SE	0.334	0.334	0.435	0.466	**0.774**				
MO	0.389	0.389	0.372	0.430	0.430	**0.760**			
ST	-0.352	-0.341	-0.250	-0.371	-0.300	-0.314	**0.827**		
AN	-0.410	-0.443	-0.403	-0.427	-0.422	-0.483	-0.341	**0.760**	
BI	0.460	0.550	0.534	0.450	0.477	0.516	-0.410	-0.519	**0.770**

### Structural model

Table 4 shows the results of the model-fit indices for a structural model. The absolute fit index is represented by X^2^/df, while the incremental fit index includes RMSEA, GFI, NFI, IFI, CFI, PNFI, PCFI, and PGFI. The X^2^/df value obtained is 2.588, below the recommended threshold of 3. The RMSEA value is 0.037, indicating an excellent fit to the model, as it is below the recommended threshold of 0.08. The GFI, NFI, IFI, and CFI values are above the recommended threshold of 0.90, indicating a good fit. The PNFI, PCFI, and PGFI values are also above the recommended threshold of 0.50, showing a good balance between model accuracy and parsimony. Based on these fit indices, the structural model appears to fit the observed data. Therefore, it can be recommended as a suitable model for the data under consideration.

**Table 4:** Model fit index

Fit Index	Absolute fit index			Incremental fit index			Parsimonious-fit index		
	**X**^**2**^**/df**	**RMSEA**	**GFI**	**NFI**	**IFI**	**CFI**	**PNFI**	**PCFI**	**PGFI**
Structural Model	2.588	0.037	0.947	0.940	0.963	0.962	0.805	0.824	0.76
Recommendation	< 3	< 0.08	≥ 0.90	≥ 0.90	≥ 0.90	≥ 0.90	> 0.50	> 0.50	> 0.50

**Fig. 3:**
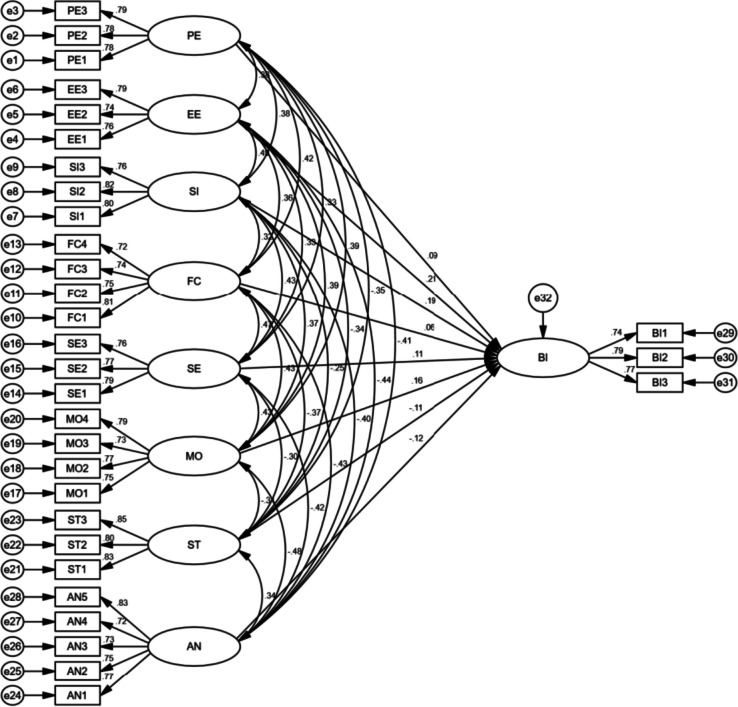
Structural model testing

In this study, the researchers used SEM path analysis to analyze the proposed hypotheses in the developed model, as shown in Fig. 3. Table 5 contains the results of the hypothesis testing. Of the eight hypotheses tested, seven were supported, and one was rejected. The study finds indicated that PE (β = 0.093, *p* < 0.01), EE (β = 0.206, *p* < 0.001), SI (β = 0.192, *p* < 0.001), SE (β = 0.111, *p* < 0.05), and MO (β = 0.16, *p* < 0.001) had a significant positive impact on BI, supporting hypotheses H1, H2, H3, H5, and H6. The study also found that the factors ST (β = -0.11, *p* < 0.001) and AN (β = -0.125, *p* < 0.001) factors were significant in affecting BI, supporting both H7 and H8. However, the effect of FC (β = 0.061, *P* > 0.01) on BI was negative but insignificant, so H4 was rejected. In general, the study supports the relationship between PE, EE, SI, SE, MO, ST, and AN constructs and BI, providing valuable information for researchers and practitioners to understand the factors that influence BI.

**Table 5:** Summary of path estimates

H#	Hypothesis	Estimate	SE	CR	*P*	Supported
H1	PE → BI	0.093	0.036	2.618	^**^	YES
H2	EE → BI	0.206	0.042	5.326	^***^	YES
H3	SI → BI	0.192	0.041	5.097	^***^	YES
H4	FC → BI	0.061	0.03	1.65	0.099	NO
H5	SE → BI	0.111	0.039	2.968	^*^	YES
H6	MO → BI	0.16	0.037	4.354	^***^	YES
H7	ST → BI	-0.11	0.029	3.442	^***^	YES
H8	AN → BI	-0.125	0.036	3.406	^***^	YES

^*^p < .05, ^**^p < .01, ^***^p < .001

## Discussion

This study used the UTAUT framework to determine what factors are associated with medical students' intentions to use Rain Classroom. A structural model was created to analyze the causal relationships among several variables: PE, EE, SI, FC, SE, MO, ST, AN, and BI. A survey questionnaire was administered to gather empirical data. The results indicated that the use of Rain Classroom, PE, EE, and SE was positively affected, while AN and ST had negative effects. FC did not show any significant influence.

The results indicated that the student's behavioural intentions to use Rain Classroom were significantly positively impacted by PE. Students find Rain Classroom a learning experience that helps them complete things more effectively. As a result, this increases student output and improves their performance. [55]. This outcome was in line with the previous studies. [8, 30, 33, 56].

According to our study, EE significantly affected students' behavioural intentions to use Rain Classroom. Evidence from a variety of research shows that EE is a predictor of future technology adoption, particularly in the realms of mobile learning and services. [8]. The results of other studies confirm this finding [57–60]. Therefore, this finding indicates that the Rain Classroom was easy for students to use.

SI had a favourable effect on students' intentions to use Rain Classroom, it was discovered. This result is consistent with previous findings [30, 61], which also showed that SI positively affects the use of interactive online learning in virtual face-to-face technology [62]. Teachers are crucial in promoting technology use for students' learning, and the sociocultural environment may encourage and support students in using Rain Classroom.

FC did not significantly affect the students' behavioural intention to use Rain Classroom. Some previous research [9, 57, 63] obtained similar findings. However, we believe that both software and hardware are crucial factors that can influence students' intentions to use Rain Classroom. It is important to provide students with resources, including the fast internet and powerful computers, to effectively utilize the technological platform. In addition, students should have sufficient access to information and resources to make the most of the platform. Successful implementation of Rain Classroom necessitates good governance in higher education and meticulous planning.

This study showed that SE positively impacted students' behavioural intentions to use Rain Classroom. The most influential factor affecting acceptance intention is SE. The more self-efficacy students have, the more confident they are in their ability to address problems in the Rain Classroom process, and they are more likely to accept Rain Classroom. This result is consistent with previous findings [1, 56, 61]. Therefore, it is very important to train students to be able to operate the Rain Classroom.

Based on this study's findings, MO positively impacted students' intentions to use Rain Classroom. According to A Kaplan and ML Maehr [64], students who are intrinsically and extrinsically motivated tend to perform better academically. The intention to behave in a certain way is influenced by external MO. E Marlina, B Tjahjadi and S Ningsih [65] found that external factors such as parental involvement, student MO, learning strategies, and socioeconomic status (SES) can affect student behaviour in the teaching and learning process, which is consistent with the findings of several other studies [64, 66–68].

This research indicated that AN had a negative impact on students' behavioural intentions to use Rain Classroom. The more anxious a student is, the less willing he or she is to persist in the Rain Classroom. These confirm the findings of other studies [30, 69–71]. This finding and those of similar studies have acknowledged that student proficiency is improved due to reinforced confidence due to the effective reduction in the negative effect of AN due to a more comfortable environment provided for students by web-based instruction technology.

In this study, ST had a negative effect on students' intentions to use Rain Classroom. The main findings show that ST was critical to the behavioural intention to accept e-learning [72]. Rain Classroom has made an effort to improve the mental well-being of students in the digital learning environment. There is no ST shouldered by the students or teachers relevant to learning to use WeChat or PowerPoint.

### Limitations

The present study has several limitations that require further investigation. First, the findings may not be generalizable due to the limited sample size of only five medical universities in China. Future research should aim to collect data from a more diverse range of universities in China and other countries to increase the validity and generalizability of the findings. Second, while the study adds four new variables based on the UTAUT model, there are likely more factors influencing students' intention to use Rain Classroom. Therefore, future research should adopt other variables to uncover more elements that influence medical students' intentions to use Rain Classroom. Third, this study relied solely on a cross-sectional survey conducted once, which may not provide a comprehensive understanding of the subject matter. It is recommended that future studies employ a range of data collection techniques, including interviews, qualitative research, and longitudinal studies, to gain a deeper insight into the determinants of medical students' willingness to utilize Rain Classroom. Finally, the research focuses primarily on a specific online learning system (Rain Classroom), so caution should be taken when generalizing the results to other technologies. Students' perceptions and experiences may differ when adopting different types of electronic learning. Therefore, future studies should duplicate this finding using various e-learning platforms, including Google Meet, Moocs, and m-learning.

## Conclusion

The study indicate that the UTAUT-based model effectively identifies factors that influence students' intention to use the Rain Classroom in higher medical education. The research expanded the UTAUT model by including constructs of self-efficacy, motivation, stress, and anxiety. Performance expectancy, effort expectancy, social influence, self-efficacy, and motivation positively impacted students' intention to use Rain Classroom, while stress and anxiety had a negative impact. Facilitating conditions did not positively affect medical students' behavioural and intention to use Rain Classroom, as per the study. The literature review revealed that these findings align with some other research. This study can be a beneficial resource for future Rain Classroom research. The results suggest that addressing students' psychological attitudes regarding new technologies and offering social context support are essential for successfully implementing Rain Classroom. Furthermore, we believe these findings could be applied to other universities in China or elsewhere.

### Supplementary Information

**Additional file 1: Appendix 1. **Please choose the appropriate response that reflects your opinion for each of the following statements.

## Data Availability

The datasets used and analysed during the current study available from the corresponding author on reasonable request.
