# Supplementary material for: Factors affecting medical students’ intention to use Rain Classroom: a cross-sectional survey
Source: BMC Med Educ. 2024 Jan 24;24:86. doi: 10.1186/s12909-024-05037-6 (PMC10807362; doi:10.1186/s12909-024-05037-6)
Supplement: Supplementary file 1 — Additional file 1: Appendix 1. Please choose the appropriate response that reflects your opinion for each of the following statements. [file 12909_2024_5037_MOESM1_ESM.docx]

Appendix 1

| Please choose the appropriate response that reflects your opinion for each of the following statements.①Strongly agree,②Agree, ③Neutral,④Disagree, ⑤Strongly disagree. Strongly agree is assigned 5 points, agree is assigned 4 points, neutral is assigned 3 points, disagree is assigned 2 points, and strongly disagree is assigned 1 point. Likert-scale 1=strongly disagree, 2=disagree, 3=neutral, 4=agree, 5=strongly agree | | | | | | | |
| --- | --- | --- | --- | --- | --- | --- | --- |
| Construct | CODE | Statements | 1 | 2 | 3 | 4 | 5 |
| Performance Expectancy  (adapted from Venkatesh  et al., 2003) | PE1 | Using Rain Classroom would enable me to accomplish learning tasks quickly. |  |  |  |  |  |
|  | PE2 | Using Rain Classroom would enhance my effectiveness in learning. |  |  |  |  |  |
|  | PE3 | Using Rain Classroom would improve my learning performance. |  |  |  |  |  |
| Effort Expectancy  (adapted from Venkatesh  et al., 2003) | EE1 | Learning to operate Rain Classroom would be easy for me. |  |  |  |  |  |
|  | EE2 | It would be easy for me to become skillful at using Rain Classroom. |  |  |  |  |  |
|  | EE3 | I will soon adapt to Rain Classroom. |  |  |  |  |  |
| Social Influence  (adapted from Venkatesh et al.,  2003) | SI1 | My use of Rain Classroom is influenced by my classmates. |  |  |  |  |  |
|  | SI2 | My use of Rain Classroom is influenced by my teachers. |  |  |  |  |  |
|  | SI3 | In general, my university has supported the use of Rain Classroom. |  |  |  |  |  |
| Facilitating conditions  (adapted from Venkatesh  et al., 2003) | FC1 | I have the resources necessary to use Rain Classroom. |  |  |  |  |  |
|  | FC2 | I have the knowledge necessary to use Rain Classroom. |  |  |  |  |  |
|  | FC3 | Rain Classroom is compatible with other systems I use. |  |  |  |  |  |
|  | FC4 | A specific person (or group) is available for assistance with the difficulties met in Rain Classroom. |  |  |  |  |  |
| Self-efficacy  (adapted from Lee and Mendlinger, 2011) | SE1 | By using Rain Classroom, I believe I will be able to achieve most of the goals specified by my tutor/teacher. |  |  |  |  |  |
|  | SE2 | By using Rain Classroom, I am sure that I can complete the learning tasks even if they are difficult. |  |  |  |  |  |
|  | SE3 | Through Rain Classroom, I think I can get results that are important to me. |  |  |  |  |  |
| Motivation   (adapted from Beluce and Oliveira, 2015) | MO1 | I am finding that Rain Classroom is very easy. |  |  |  |  |  |
|  | MO2 | I have found that Rain Classroom is convenient. |  |  |  |  |  |
|  | MO3 | I have found that Rain Classroom is useful for my studies. |  |  |  |  |  |
|  | MO4 | Rain Classroom helped me become more motivated to study. |  |  |  |  |  |
| Stress  (adapted from et al., 2021) | ST1 | I have faced several difficulties at the time of taking classes in Rain Classroom as it has not been demonstrated to me. |  |  |  |  |  |
|  | ST2 | Studying online for a long time, I worry about my personal health (becoming depressed) |  |  |  |  |  |
|  | ST3 | I have been having issues being social, as there is no physical interaction between the teachers and the students or between classmates. |  |  |  |  |  |
| Anxiety  (adapted from Yoshida et al., 2016) | AN1 | I am worried about whether I can properly operate Rain Classroom. |  |  |  |  |  |
|  | AN2 | I am worried about whether I can accomplish the learning objective via Rain Classroom. |  |  |  |  |  |
|  | AN3 | I feel apprehensive about learning via Rain Classroom. |  |  |  |  |  |
|  | AN4 | I am nervous while participating in group discussions. |  |  |  |  |  |
|  | AN5 | I am worried about my achievements in online cooperative learning. |  |  |  |  |  |
| Behavioral Intention (adapted from Venkatesh  et al., 2003) | BI1 | I think using Rain Classroom is a very good way to promote learning. |  |  |  |  |  |
|  | BI2 | I would like to recommend the use of Rain Classroom to others. |  |  |  |  |  |
|  | BI3 | I am willing to use Rain Classroom more frequently in the future. |  |  |  |  |  |
